# Supplementary material for: Novel CRYGC Mutation in Conserved Ultraviolet-Protective Tryptophan (p.Trp131Arg) Is Linked to Autosomal Dominant Congenital Cataract
Source: Int J Mol Sci. 2023 Nov 22;24(23):16594. doi: 10.3390/ijms242316594 (PMC10706789; doi:10.3390/ijms242316594)
Supplement: Supplementary file 1 [file ijms-24-16594-s001.zip › ijms-2605471-supplementary.pdf]

Table S1. Cataract-associated gene list used for filtering of WES data.

ABCA3	COL4A5	ETFDH	HIP1	MYH9	PEX7	SRD5A3
ABCD3	COL7A1	EYA1	HMX1	MYOC	PIGY	STX3
ABHD12	CPAMD8	EZR	HSF4	NACC1	PITX2	TAF1A
ADAM9	CRYAA	FAM126A	HSPG2	NAT8	PITX3	TAPT1
ADAMTS18	CRYAB	FAR1	IARS2	NCOA6	POLG	TDRD7
ADAMTSL4	CRYBA1	FBN1	IDO1	NECAP2	POMGNT1	TFAP2A
AGK	CRYBA2	FKRP	IKBKG	NECTIN3	POMT1	TMCO3
ALDH18A1	CRYBA4	FKTN	INPP5K	NEU1	POMT2	TMEM70
ANK2	CRYBB1	FLNB	ITM2B	NF2	PQBP1	TNPO1
ARSL	CRYBB2	FOXE3	JAM3	NHS	PRDX5	TRAPPC11
ATAD3A	CRYBB3	FTL	KCNA4	NOD2	PROX1	TRNT1
B3GLCT	CRYGA	FYCO1	KCNAB1	NR2E3	PRX	TRPM3
BCOR	CRYGB	FZD4	KCNJ13	NRCAM	PTEN	TUBA1A
BEST1	CRYGC	GALE	KLRG1	OAT	PTH	TUBB2A
BFSP1	CRYGD	GALK1	LARGE1	OCRL	PXDN	UCHL1
BFSP2	CRYGS	GALT	LEMD2	OGG1	RAB3GAP1	UNC45B
BIN3	CTDP1	GCM2	LIM2	OPA1	RECQL4	VCAN
BRD4	CYP27A1	GCNT2	LMX1B	OPA3	RGS6	VIM
BUB1B	CYP51A1	GDF3	LONP1	OTX2	RIC1	VLDLR
CANX	DHCR7	GEMIN4	LOXL3	P3H2	RRAGA	VSX2
CAV1	DMPK	GFER	LRP2	PANK4	RYR1	WDR36
CC2D2A	DNASE2B	GJA1	LRP5	PARK7	SALL4	WDR87
CCNP	DNM2	GJA3	LRP5L	PAX6	SC5D	WFS1
CDC25B	DNMBP	GJA8	LSS	PEX1	SEC23A	WNT3
CHD7	DOCK5	GJB6	MAF	PEX10	SIL1	WRN
CHMP4B	DYNC1H1	GLA	MAFA	PEX11B	SIPA1L3	XRCC1
CLPB	EFNA5	GNAS	MAN2B1	PEX12	SIX5	XYLT2
CNBP	EIF2B2	GNPAT	MED12	PEX13	SLC16A12	YWHAE
CNGB3	EPG5	GPR161	MED13	PEX14	SLC33A1	ZNF350
COL11A1	EPHA2	GPX1	MIP	PEX16	SLC4A4	
COL18A1	ERCC2	GSTM1	MIPEP	PEX2	SLC7A8	
COL2A1	ERCC6	GSTT1	MIR184	PEX26	SORD	
COL4A1	ERCC8	GUCY2D	MMP1	PEX3	SOX1	
COL4A2	ESCO2	HCCS	MVK	PEX5	SPARC	
